# Graphene Oxide with Controlled Content of Oxygen Groups as a Filler for Polymer Composites Used for Infrared Radiation Shielding

**DOI:** 10.3390/nano10010032

**Published:** 2019-12-21

**Authors:** Marta Mazurkiewicz-Pawlicka, Maksymilian Nowak, Artur Malolepszy, Andrzej Witowski, Dariusz Wasik, Yi Hu, Leszek Stobinski

**Affiliations:** 1 Faculty of Chemical and Process Engineering, Warsaw University of Technology, Warynskiego 1, 00-645 Warsaw, Poland; msf.nowak@gmail.com (M.N.); artur.malolepszy@pw.edu.pl (A.M.); 2 Faculty of Physics, Institute of Experimental Physics, University of Warsaw, Pasteura 5, 02-093 Warsaw, Poland; Andrzej.Witowski@fuw.edu.pl (A.W.); dariusz.wasik@fuw.edu.pl (D.W.); 3 Department of Materials Engineering, Tatung University, Taipei 104, Taiwan; huyi@gm.ttu.edu.tw; 4 Polski Bazalt S.A., Weteranow 157, 05-250 Radzymin, Poland; lstob50@hotmail.com; 5 NANOMATERIALS LS (www.nanomaterials.pl), Wyszogrodzka 14/38, 03-337 Warsaw, Poland

**Keywords:** graphene oxide, polymer nanocomposites, reduced graphene oxide, infrared shielding

## Abstract

Infrared (IR) shielding materials are commonly used for different applications, such as smart windows or optical filters. Infrared radiation is responsible for about 50% of the energy coming from the sun. During a hot summer or cold winter a lot of energy is needed to keep the optimal temperature inside buildings and means of transport. To reduce the heat transmission and save energy IR shielding materials can be used as coatings made of polymer composites. Graphene oxide (GO) and its reduced forms have interesting IR absorption properties and might be used as a filler in a polymer matrix for IR shielding applications. Graphene oxide can be reduced by different methods. Depending on the reduction method reduced graphene oxide (rGO) with a different content of oxygen can be obtained exhibiting different properties. In this work we propose new polymer nanocomposites with poly(vinyl alcohol) as the matrix and 0.1 wt.% addition of graphene materials with different oxygen content to be used for IR shielding applications. The results show that the properties of the graphene filler strongly influence the infrared shielding properties of the obtained nanocomposites. The best IR shielding properties were obtained for the composites where rGO with the lowest oxygen content was used.

## 1. Introduction

Infrared shielding materials can be used in different applications, such as smart windows, solar collectors or optical filters [1,2,3]. Near-infrared (NIR) radiation is responsible for around 50% of solar energy and heat resulting from it [4,5]. Thermal energy coming from NIR radiation can be responsible for heat accumulation inside buildings or cars. During summer, a lot of energy is needed to cool down the indoor spaces and during winter energy is consumed for keeping the heat inside. To save the energy NIR shielding materials can be used as coatings in buildings and cars to reduce the heat transmission through their outer surfaces. These coatings can be divided into inorganic materials (such as heat-reflective glasses) [1,3,6,7] and polymer composites with inorganic fillers (such as indium tin oxide-ITO, antimony tin oxide-ATO, lanthanum hexaboride-LaB_6_, tungsten oxide-WO_3_ nanoparticles) [4,8,9,10,11]. The fillers should possess high absorption or high reflectance of infrared radiation. Usually, for polymer composites good infrared (IR) shielding is obtained with an addition of few wt.% of the filler (around 1–5 wt.%) which can change the mechanical and thermal properties of the used polymer, not to mention the price of the material obtained [4,8,9,11]. Therefore new materials used as fillers in the polymer composites are sought.

Poly(vinyl alcohol)-PVA is a synthetic water soluble polymer with great mechanical and thermal properties and can easily form films [12,13]. PVA is often used in biomedical, food industry, adhesives, coatings and membrane applications [12,14,15,16,17]. PVA has a lot of hydroxyl groups which show high infrared absorption. These properties make PVA an interesting material to be used as the polymer matrix for IR shielding nanocomposite preparation.

Graphene is a carbon allotrope with great mechanical, thermal, electrical and optical properties [18,19,20,21]. Potential applications of graphene depend on its properties which are connected to the preparation method. One of the methods is oxidizing of graphite which results in obtaining graphene oxide (GO) with many functional groups (such as epoxide, hydroxyl or carboxyl) [22,23]. Strong oxidation of graphite results in a decrease of electrical conductivity and optical absorption due to disrupted π conjugation [24]. Reduction of graphene oxide leads to partial restoration of the π conjugation and can increase the electrical conductivity and optical absorption of the material obtained (reduced graphene oxide-rGO). But depending on the oxygen atoms and their arrangement in the rGO structure (mainly on edges of defects) the material exhibits very strong infrared absorption [19]. The IR absorption properties of graphene materials were used mostly for biomedical applications, such as photothermal therapy [24,25]. Adding graphene to polymers can enhance their electrical, mechanical and optical properties [26,27,28,29]. Combining rGO possessing high IR absorption properties with PVA can result in a material with good infrared radiation shielding.

The aim of this work was to study the infrared and thermal shielding properties of obtained graphene/PVA composites. We have shown that the reduction method of graphene oxide leads to a material with different content of oxygen groups that can influence the IR shielding properties of prepared composites. Notably, our composites show good NIR shielding properties with an addition of just 0.1 wt.% of graphene filler, which is much lower (around 10–50 times) than for composites containing inorganic nanoparticles (such as ITO). This might reduce the price of the material that can be used for IR shielding coatings.

## 2. Materials and Methods

### 2.1. Graphene Oxide (GO) Preparation

Graphene oxide (GO) was prepared by a modified Hummers method [22,23]. Generally, for oxidizing 10 g of graphite powder (Acros Organics, Geel, Belgium) 230 mL of sulfuric acid (98 wt.%), 4.7 g of sodium nitrate and 30 g of potassium permanganate were used. The graphite powder was added to sulfuric acid and stirred for 30 min. Next, sodium nitrate and potassium permanganate were slowly added and the whole mixture was kept in an ice bath to keep the temperature below 10 °C. After this step the mixture was slowly heated to 35 °C and stirred for two hours. After that 100 mL of deionized water was added to the mixture and the temperature reached ~100 °C. Finally, the mixture was treated with 10 mL of 30 wt.% H_2_O_2_. The obtained slurry was kept in an ultrasonic bath for 1 h and next it was thoroughly filtered through a ceramic membrane system with 0.2 micron pore size and washed with deionized water until the pH of the filtrate reached ~7. Graphene oxide was obtained as a water suspension with the concentration of 14 mg/mL.

### 2.2. Reduction of Graphene Oxide

Graphene oxide was reduced chemically and thermally. For the first method two different reducing agents were used: citric acid and hydrazine hydrate solution. The thermal reduction was conducted at two different temperatures: 220 °C and 1000 °C.

The chemical reduction with citric acid was performed by preparing a diluted suspension of GO where 66 g of 14 mg/mL GO suspension was dispersed in 100 mL of water with the use of an ultrasonic homogenizer for 15 min. Citric acid solution was prepared by dissolving 7.25 g powder in 25 mL of water. Citric acid was then added dropwise in the diluted GO suspension and stirred in a closed bottle at 80 °C for 48 h. Next, the obtained reduced graphene oxide was filtered and rinsed with deionised water until neutral pH was reached and dried at room temperature. The obtained sample was denoted as rGO1.

Thermal reduction at lower temperature was performed by heating 62 g of 14 mg/mL GO water suspension in a laboratory dryer for 2 h at 220 °C. The obtained sample was denoted as rGO2.

Chemical reduction with hydrazine hydrate was performed by boiling 100 mL of 14 mg/mL GO suspension with 10 mL of 1M NaOH and 10 mL of hydrazine hydrate for 30 min under reflux. After that period the sample was cooled and the obtained slurry was filtrated and rinsed with deionised water until neutral pH of the filtrate was reached. The sample was dried at 130 °C. The obtained sample was denoted as rGO3.

The last sample was prepared by heating rGO3 in a quartz tube at 1000 °C in argon flow for 10 min. Obtained sample was denoted as rGO4.

### 2.3. Composite Preparation

Poly(vinyl alcohol) powder (Mw = 72,000, Avantor Performance Materials, Poland) was used to prepare the polymer composites with graphene materials used as fillers. In all the samples 0.1 wt.% of the filler was used. For the sample containing GO a 10 wt.% solution of PVA in water was prepared. A proper amount (around 180 µL) of 14 mg/mL GO suspension was added to 25 mL of prepared PVA solution and the mixture was sonicated for 10 min in an ultrasonic homogenizer (Hielsher UP400S). We poured 15 mL of the obtained mixture on a plastic Petri dish and dried it at 60 °C. The obtained composite was denoted as 0.1% GO.

The samples with reduced graphene oxide were prepared as follows: 2 mg of rGO powder were mixed with 2 g of PVA powder in a planetary ball mill (Retsch PM100) for 30 min at 400 rpm. Next, the obtained powder was dissolved in water to form a 10 wt.% solution. We then poured 15 mL of the obtained solution on a plastic Petri dish and dried it at 60 °C. The obtained composites were denoted as 0.1% rGO1, 0.1% rGO2, 0.1% rGO3 and 0.1% rGO4 depending of the graphene filler that was added.

The average thickness of the obtained polymer composites is presented in Table 1.

### 2.4. Sample Characterization

Carbon nanomaterials were analyzed with the use of different techniques, such as thermogravimetric analysis (TGA), elemental analysis (carbon, hydrogen, nitrogen, sulfur-CHNS), and Fourier transform infrared (FT-IR) spectroscopy. The composite films were analyzed with the use of FT-IR spectroscopy, ultraviolet–visible-near infrared (UV–Vis-NIR) spectroscopy and TGA analysis. For the composite films a temperature measurement was also performed with the use of a home-made equipment.

Carbon, nitrogen, hydrogen and sulfur content in graphene samples was evaluated by Flash 2000 (CHNS/O) analyzer (Thermo Scientific, Waltham, MA, USA). For each sample three measurements were performed, where 1–2 mg of the carbon material was analyzed. The analysis was conducted in a furnace at 960 °C in a helium atmosphere where oxygen was injected after 12 s. Oxygen content was calculated by subtraction of C, H, N, S and ash content (from TGA measurements) from 100%.

TGA measurements were performed in a Mettler Toledo TGA/DSC 3+ thermogravimetric analyzer at a temperature range from 30 °C to 1000 °C with 10 °C/min heating range with 30 mL/min airflow for carbon nanomaterials. For the polymer samples, the measurements were carried out at a temperature range from 25 °C to 500 °C with 10 °C/min heating range with 30 mL/min airflow.

For carbon nanomaterials, FT-IR spectroscopy in the mid-infrared (MIR) region was carried out with Nicolet iS10 (Thermo Scientific, Waltham, USA) spectrometer in attenuated total reflectance (ATR) mode on a diamond crystal and in transmission mode. For the transmission measurements, KBr pellets were prepared (0.3 wt.% of graphene material in KBr). For composite materials the samples were measured in transmission mode without any preparation.

UV–Vis-NIR spectroscopy was performed for obtained polymer composites with the use of CARY 5000 spectrometer (Agilent Technologies, Santa Clara, CA, USA) without any preparation.

**Figure 1:**
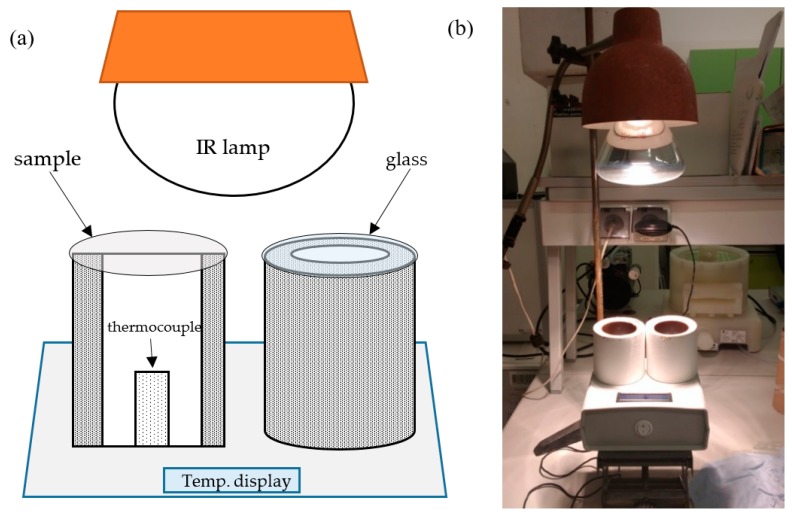
Equipment for temperature measurements: (**a**) scheme (cross-section of the measurement tube on the left), (**b**) photo.

The prepared composites were tested in a home-made equipment, where infrared lamp was irradiating two specimens (polymer sample and glass cover as a reference) and the temperature beneath was measured with a thermocouple. The plastic tubes where the temperature was measured were isolated from the surroundings by a polyurethane foam and an outer plastic tube [30]. A photo and scheme of the equipment are presented in Figure 1. In Figure 1a a cross section of the measurement tube is presented on the left. The measurement was performed as follows: the IR lamp was turned on and when the same temperature (around 37 °C) was reached in both tubes the polymer sample was placed on one tube and a glass cover on the other. The temperature was measured for 25 min in 5 min intervals. After the measurement, the lamp was turned off for the temperature in the tubes to equalize.

## 3. Results and Discussion

### 3.1. Graphene Nanomaterials Characterization

**Figure 2:**
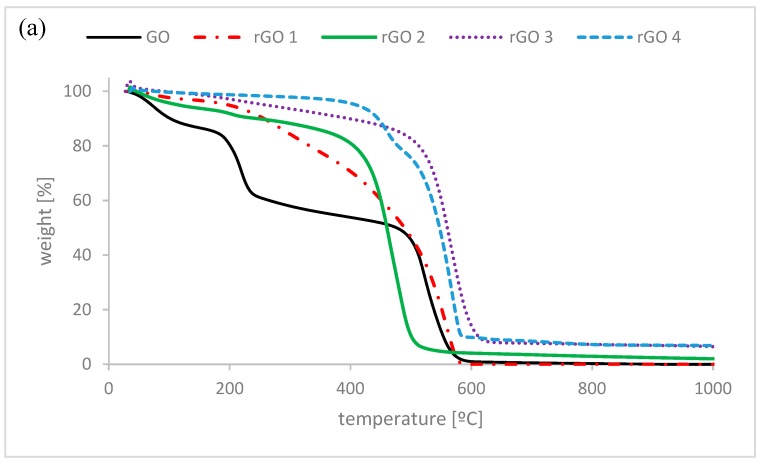

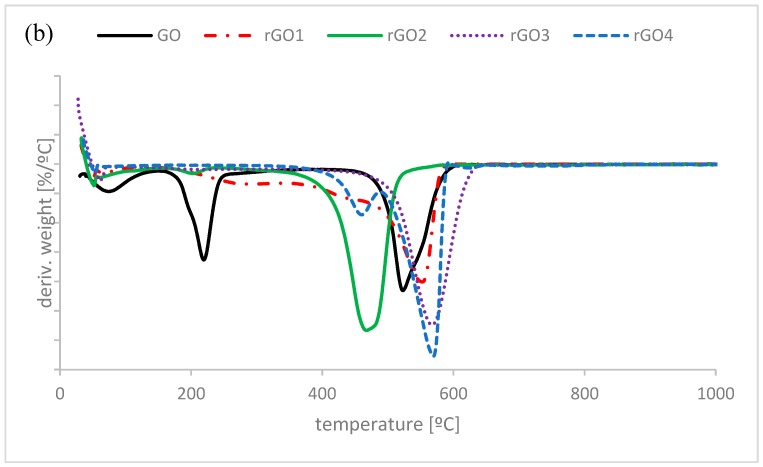
(**a**) Thermogravimetric analysis (TGA) and (**b**) derivative thermogravimetric (DTG) curves of obtained graphene materials.

Thermogravimetric measurements can give information about the thermal stability of the prepared graphene materials. It also can be used to evaluate the purity of the analyzed materials. In Figure 2 TGA and derivative thermogravimetry (DTG) curves are presented for the obtained graphene samples. The DTG graph shows three peaks for graphene oxide. The first peak corresponds to the loss of water (around 10 wt.% from TGA) present in the sample even after drying. GO starts decomposing at ~200 °C where the least stable oxygen groups are removed. It can be seen that after this process ~60 wt.% of the material is left. This suggests that GO has a lot of oxygen containing functional groups. Another thermal decomposition starts at ~500 °C after which the material is fully decomposed (residue is 0 wt.%). This might suggest that the obtained graphene oxide was very pure. For all reduced samples there is a much lower content of water present in the material compared to GO. For the sample reduced with a mild reducing agent (rGO1) decomposition starts at ~200 °C but it has less groups decomposing at this temperature comparing to GO. From 200 °C a steady decline in the sample weight is observed and the highest decomposition is observed at ~500 °C. This might suggest that some groups have been removed, but there are still a lot of oxygen functional groups present in the sample. Graphene oxide reduced at low temperature (rGO2) exhibits a small weight loss at ~200 °C. This suggests that most of the least stable oxygen groups from GO were removed, but some of them are still present. Another decomposition for the rGO2 starts at ~400 °C and there are some impurities left (results presented in Table 2). Graphene oxide reduced with hydrazine hydrate (rGO3) shows high thermal stability comparing to previous samples. There is around 20 wt.% loss up to 500 °C where the rGO3 sample starts decomposing. This might suggest that most of the oxygen groups were reduced with the use of a strong reducing agent. High temperature treatment of the sample reduced by hydrazine (sample rGO4) is effective in removing the least stable oxygen functional groups. Two peaks can be seen in the DTG graph, giving information about two-step decomposition of rGO4 sample. The first step of thermal decomposition of the sample starts at ~400 °C the second step starts at ~500 °C and some impurities are left. The TGA results show that the reduction processes used for graphene oxide never removed all of the oxygen groups in GO but the best results were obtained for samples reduced with a strong reducing agent and at high temperature. For both samples that were thermally reduced (rGO2 and rGO4) decomposition of some groups starts at a lower temperature (~400 °C) compared to chemically reduced materials. This might suggest a different mechanism of reduction of the samples leading to formation of different functional groups.

Results obtained from elemental analysis are presented in Table 2. It can be seen that graphene oxide is rich in oxygen (~47 wt.%), suggesting a lot of functional groups present in the material. Sulfur probably comes from the preparation procedure. Reduction of GO with a weak reducing agent (rGO1) or at a low temperature (rGO2) results in a slight loss of oxygen (to ~30 wt.%) and enrichment of the sample in carbon. These results indicate that the used methods can end in obtaining graphene materials with a low degree of reduction and still rich in oxygen. Using strong reducing agent (rGO3) or high temperature (rGO4) results in a significant removal of the oxygen groups (leaving around 8 wt.%) and high carbon content (more than 80 wt.%). In rGO3 sample there is around 2 wt.% of nitrogen which comes from hydrazine, but it is removed after high temperature treatment. The results show that even strong reducing agents or high temperature leave some oxygen groups present in the carbon structure.

**Figure 3:**
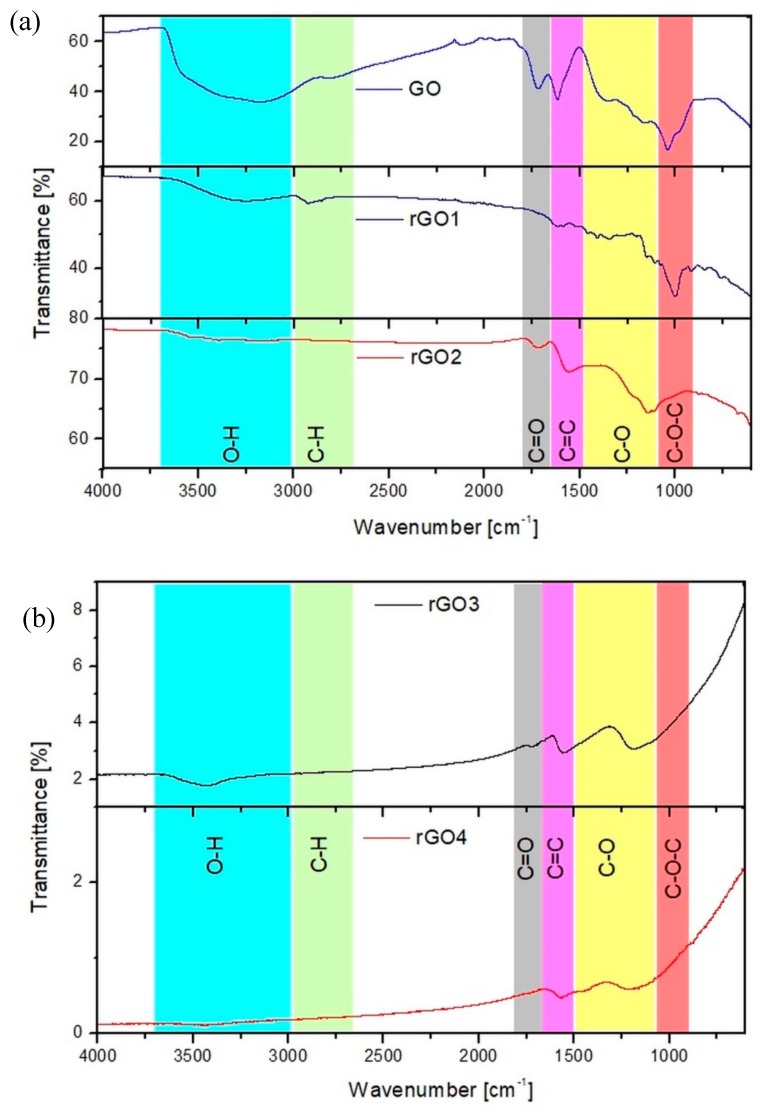
Fourier transform infrared (FT-IR) spectra of graphene materials in (**a**) attenuated total reflectance (ATR) and (**b**) transmission modes.

The chemical composition of the obtained graphene materials was also analyzed with the use of the FT-IR spectrometer. For the samples with many functional groups (GO, rGO1 and rGO2) the measurements were performed by the ATR technique. Due to a strong absorption of IR radiation for the rGO3 and rGO4 samples it was necessary to perform the measurements in transmission mode where KBr pellets with 0.3 wt.% of the sample were prepared. The results are presented in Figure 3 with possible functional groups identification.

As can be seen, graphene oxide has a lot of oxygen groups in its structure, especially O-H (from water present in the sample as well as from hydroxide groups), C-O and C-O-C groups. For graphene oxide a strong peak can be observed at around 1600 cm^−1^, which is usually assigned to C = C bonding, but in the case of GO it most likely arises from water present in the material [31]. For the rGO1 sample a small broad peak is still visible at ~3500 cm^−1^ and a strong peak at ~1000 cm^−1^ is still present after the reduction. Other peaks have been significantly reduced, but C-H bonds are visible in this sample. These results indicate that after reduction with a weak reducing agent the water from the sample was removed and most of the oxygen groups were removed. For the sample reduced at low temperature (rGO2) the broad peak at ~3500 cm^−1^ is significantly decreased, the peak at ~1700 cm^−1^ also decreased and the peak at ~1000 cm^−1^ is shifted to higher wavenumbers. These results might suggest that for rGO2 sample the water and hydroxyl, epoxy and carbonyl groups were removed from the sample, but the sample still has a lot of functional groups present in the structure. For samples with lower oxygen content (rGO3 and rGO4) it can be seen that most of the peaks disappeared in the spectra. The peak at ~3500 cm^−1^ is present, which might come from KBr pellets or from hydroxyl groups that were left in the carbon structure. The high reduction degree of the samples can be confirmed by a strong peak at ~1600 cm^−1^, which corresponds to C = C bonds. In both samples some peaks from C-O bonds are visible and in rGO3 sample a small peak corresponding to C = O bonds is present. These results suggest that rGO3 and rGO4 samples have still some oxygen groups present in their structure. These results show also that using different reduction methods can result in material with different functional groups present in the carbon structure. This is an important factor affecting the optical properties of reduced graphene oxide.

### 3.2. Polymer Nanocomposites Characterisation

The nanocomposites were tested for their IR shielding efficiency. The tests were performed by spectroscopic methods (transmittance of the radiation was measured in UV–Vis-NIR and MIR ranges) and by temperature measurements on a home-made equipment (Figure 1). Spectroscopic methods gave information about how much of the radiation is transmitted through a prepared polymer sample and the temperature tests allowed to measure the effectiveness of the heat shielding of the nanocomposites.

**Figure 4:**
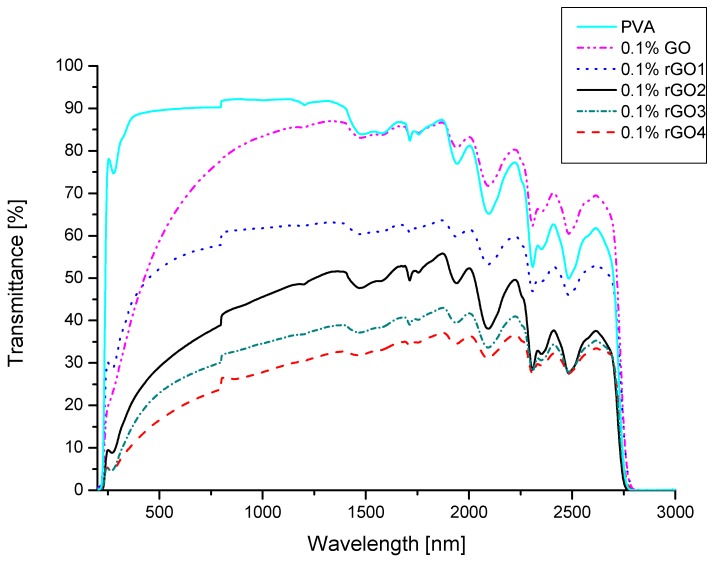
Ultraviolet–visible near infrared (UV–Vis-NIR) transmission spectra of polymer nanocomposites.

Transmittance of the UV–Vis-NIR radiation for prepared samples is presented in Figure 4. Step like structure at about 750 nm is an artefact arising from grating change. The average transmittance values obtained for the samples are listed in Table 3. Average values in different spectral regions were calculated from the following equation:

(1) $$T=\frac{\int_{\lambda_{1}}^{\lambda_{2}}T\left(\lambda \right)d\lambda }{\lambda_{2}-\lambda_{1}}$$

where *T*(*λ*) is the transmittance value at the *λ* wavelength; *λ*_1_ and *λ*_2_ are the minimum and maximum wavelength values of a spectral region, respectively [8].

The obtained results show that addition of 0.1 wt.% of graphene-based filler changes the behavior of PVA sample. Addition of graphene oxide is beneficiary for shielding UV radiation, but it doesn’t influence the properties of PVA in the NIR region. For all the samples where reduced graphene oxide materials were used as a filler transmission of UV–Vis-NIR radiation is much lower than for pure PVA. It can be seen that the best results are obtained for the sample where strongest GO reduction process occurred. This might be due to the fact, that reduced graphene oxide has high absorption of the NIR radiation [24]. It is connected to π conjugation in reduced graphene oxide structure. GO has a disrupted π conjugation due to many oxygen groups and defects present in its structure. Reduction of GO is responsible for partial restoration of the π conjugation which results in an increase of the electrical conductivity and optical absorption of the obtained material. As presented earlier different reduction methods result in rGO materials with various oxygen content and different functional groups. Weaker reduction methods lead to materials that have higher oxygen content, hence more defects present in the structure and weaker NIR absorption properties. Strong reduction methods remove more functional groups and generate higher restoration of the π conjugation which results in better NIR radiation absorption. Unfortunately, transmission in the visible region is also lower for samples with rGO fillers, but they still are transparent enough to see through them with a tint of gray. This means that the prepared materials could be used where there is no necessity for high transparency of the visible spectrum, e.g., building facades or rooftops.

**Figure 5:**
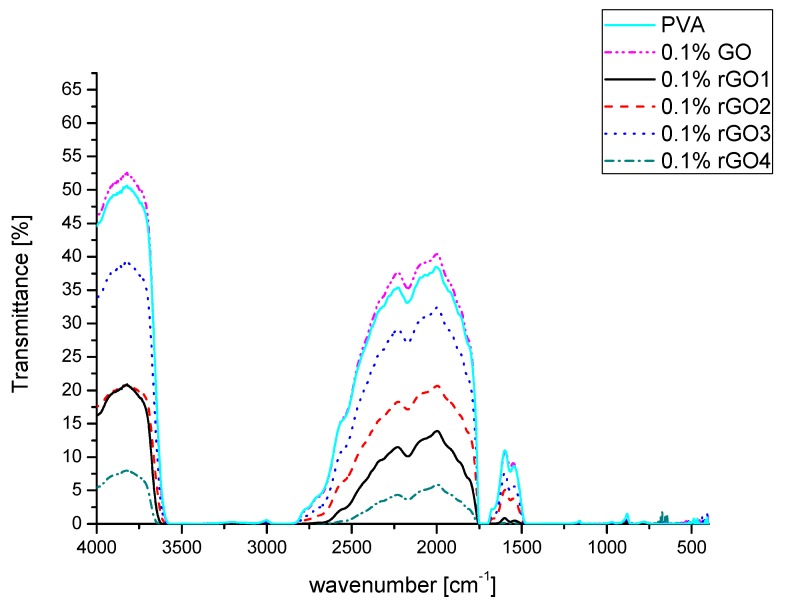
Mid-infrared (MIR) transmission spectra of polymer nanocomposites.

In Figure 5 transmission of mid-infrared radiation of prepared polymer composites is presented and the average transmittance values obtained for the samples are listed in Table 4 (calculated from Equation (1)). Similar to UV–Vis-NIR spectroscopy, better results are obtained for samples where reduced graphene oxide was used as a filler. What is interesting is that the best results were obtained for a different sample (rGO3) and rGO4 sample was third best. This behavior of the samples is probably connected to different oxygen functional groups and defects present after different reduction methods of the rGO materials used. It was shown that a high absorption in MIR is obtained for reduced graphene oxide having aggregated oxygen atoms at the edges of defects [32]. Using different reduction methods might lead to formation of different oxygen atoms’ arrangement in the carbon structure resulting in their ability to absorb MIR radiation.

**Figure 6:**
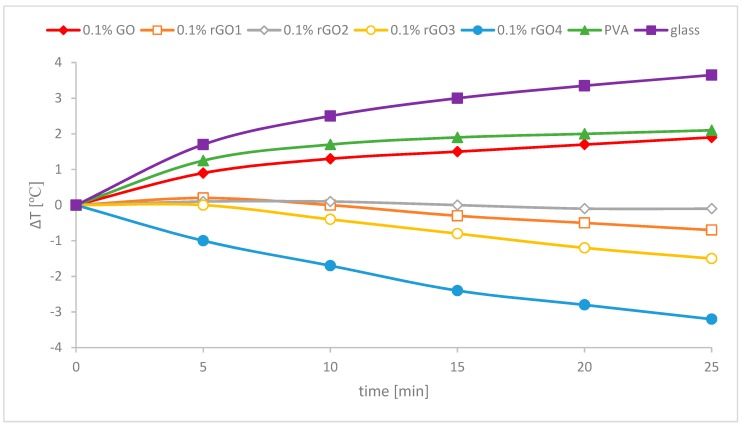
Temperature measurements of polymer nanocomposites.

The results of temperature tests performed for the obtained composites are presented in Figure 6. The glass sample is presented as a reference. For each sample the temperature is presented as ΔT, which can be expressed as the following equation:

(2) $$\Delta T=T_{x}-T_{0}$$

where *T*_0_ is the temperature in the tube at the start of the measurement (around 37 °C) and *T_x_* is the temperature after 5, 10, 15, 20 or 25 min.

It can be seen that all of the prepared samples have better temperature shielding compared to glass. After heating the samples with the IR lamp, the temperature inside the tube for glass cover rises the fastest (over 3.5 °C after 25 min). For pure PVA and the composite with graphene oxide the temperature also rises, but slower (around 2 °C after 25 min). This means that PVA and PVA/GO samples absorb more IR radiation than a thicker glass cover. But better results were obtained when rGO was added to the polymer matrix, when the temperature decreased after 25 min for all samples (from 0.1 to 3.2 °C). Better heat shielding properties of the nanocomposite arise when a filler with good IR absorption or reflectance is added to the polymer. Reduction of GO leads to partial restoration of the π conjugation in the carbon structure increasing the absorption of the IR radiation by rGO. The best results were obtained for the sample containing strongly reduced graphene oxide (rGO4), where the temperature almost instantly decreased. These results are in good agreement with the UV–Vis-NIR spectroscopy measurements. The good heat shielding properties in obtained polymer composites come mainly from absorption of the IR radiation by the addition of rGO as a filler and depend on the quality and quantity of oxygen functional groups present in the carbon structure.

**Figure 7:**
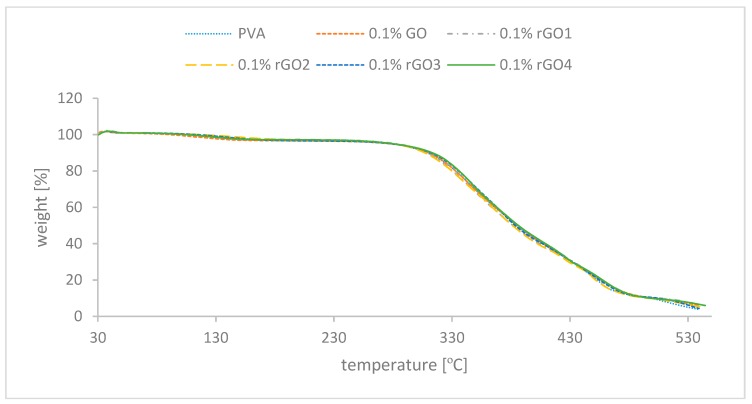
Thermogravimetric measurements of polymer nanocomposites.

TGA measurements were performed to see if the addition of graphene materials to PVA matrix changed the thermal resistance of the used polymer. The results are presented in Figure 7. The results obtained show that there is no significant change in the behaviour of the polymer after adding the nanofillers. This is a good indicator for future applications, where the polymer will have unchanged thermal properties with additional IR shielding.

## 4. Conclusions

In this paper we prepared polymer nanocomposites based on poly(vinyl alcohol) with the addition of graphene oxide materials as a filler which can be used for infrared radiation shielding. Graphene oxide was reduced using chemical (with citric acid or hydrazine hydrate) and thermal (200 °C or 1000 °C) reduction methods. Different contents of oxygen groups on graphene materials were obtained. Samples that were treated at higher temperature or with a stronger reducing agent had a lower content of oxygen (around 8–9 wt.%), where milder reduction resulted in around 30 wt.% of oxygen in the material. Composites prepared with the addition of strongly reduced graphene oxide have better IR shielding properties, especially in the NIR region. High absorption of IR radiation by reduced graphene oxide materials is mainly responsible for the shielding properties of the composites obtained. Temperature tests show that the best sample can lower the temperature by over 3 °C after 25 min. TGA measurements show that addition of 0.1 wt.% of the graphene fillers does not significantly influence the behavior of the PVA matrix. Comparing to other materials (e.g., ITO nanoparticles) similar results of NIR transmittance are obtained for much lower filler concentration (around 1–5 wt.% ITO vs. 0.1 wt.% rGO), which might result in a lower price of the final product.

## Figures and Tables

**Table 1:** Average thickness of the obtained polymer composites.

Sample Name	Thickness [mm]
0.1% GO	0.229 ± 0.019
0.1% rGO1	0.256 ± 0.047
0.1% rGO2	0.266 ± 0.049
0.1% rGO3	0.337 ± 0.033
0.1% rGO4	0.381 ± 0.051

**Table 2:** Elemental composition (carbon, hydrogen, nitrogen, sulfur and oxygen-CHNSO) and residue from TGA measurements for the obtained graphene materials.

Sample	Elemental Content					
	TGA [wt.%]	C [wt.%]	H [wt.%]	N [wt.%]	S [wt.%]	O [wt.%]
**GO**	0.00	48.98	2.18	0.00	1.03	47.20
**rGO1**	0.00	67.56	1.62	0.00	0.00	30.82
**rGO2**	1.88	67.48	0.87	0.00	0.00	29.77
**rGO3**	6.35	82.70	0.68	1.66	0.00	8.61
**rGO4**	6.78	83.21	0.64	0.00	0.53	8.84

**Table 3:** Average transmittance of UV–Vis-NIR radiation for prepared polymer samples.

Sample	UV (200–400 nm) [%]	Vis (400–800 nm) [%]	NIR (800–2600 nm) [%]
**PVA**	67.57	89.81	80.19
**0.1% GO**	26.23	65.54	79.88
**0.1% rGO 1**	31.01	54.23	59.03
**0.1% rGO 2**	12.31	32.51	45.65
**0.1% rGO 3**	8.29	25.41	36.71
**0.1% rGO 4**	6.59	19.14	31.92

**Table 4:** Average transmittance of MIR radiation for prepared polymer samples.

Sample	Transmittance [%]
PVA	12.25
0.1% GO	12.69
0.1% rGO 1	9.54
0.1% rGO 2	3.81
0.1% rGO 3	1.38
0.1% rGO 4	5.58
